# Effect of Training and Detraining in the Components of Physical Fitness in People Living With HIV/AIDS

**DOI:** 10.3389/fphys.2021.586753

**Published:** 2021-09-22

**Authors:** Juliany de Souza Araujo, Rafaela Catherine da Silva Cunha de Medeiros, Tatiane Andreza Lima da Silva, Danielle Coutinho de Medeiros, Jason Azevedo de Medeiros, Isis Kelly dos Santos, Phelipe Wilde, Breno Guilherme de Araújo Tinoco Cabral, Radamés Maciel Vitor Medeiros, Paulo Moreira Silva Dantas

**Affiliations:** ^1^Department of Physical Education, Federal University of Rio Grande do Norte, Natal, Brazil; ^2^Health Sciences Center, Federal University of Rio Grande do Norte, Natal, Brazil; ^3^Department of Physical Education, State University of Rio Grande do Norte, Mossoró, Brazil

**Keywords:** detraining, exercise, HIV, physical fitness, cardiorespiratory fitness

## Abstract

The aim of the study was to evaluate the effect of training and detraining on the physical fitness components of people living with HIV/AIDS (PLHA). The study was characterized as experimental with a sample composed of 21 people divided into two groups: 11 volunteers (PLHA, 46.9 ± 8.0 years, 63.8 ± 12.7 kg, 161.7 ± 8.7 cm, 7 men, and 4 women), using antiretroviral therapy (ART) and 10 people without HIV/AIDS in the control group (CG, 43.8 ± 13.8 years, 75.2 ± 11.2 kg, 163.3 ± 7.8 cm, 3 men, and 7 women), with the same average age and level of physical activity. The intervention, applied to both groups, consisted of combined training for 15 weeks, followed by detraining for 5 weeks. Before and after the training and detraining period the following parameters were evaluated: body composition by dual energy radiological absorptiometry (DXA), cardiorespiratory fitness by ergospirometer, and strength of upper and lower limbs by isometric dynamometer. The results show the effect of the intervention moments on the strength and oxygen consumption variables (time factor), considering the two study groups. Regarding the analysis of the interaction (group vs. time), there was a significant effect on the isometric extension strength of the left (*p* = 0.019) and right (*p* = 0.030) knees, with training (left: 10.4%; right: 12.4%) and detraining (left: −10.8%; right: −12.1%) effect in PLHA, when compared with the control group (left: 8.1 and 3.9%, respectively; right: 11.5 and −0.2%, respectively). In addition, there was a significant interaction on ventilatory threshold 1 (*p* = 0.002), indicating a significantly greater increase with training (27.3%) and decrease with detraining (−22.7%) in the PLHA group compared with the Control group (19.9 and −6.7%, respectively). In conclusion, combined training and the subsequent period of detraining caused similar responses in body composition, isometric strength, and cardiorespiratory fitness of PLHA and CG, except for the extensor strength of the lower limbs and ventilatory threshold 1, which presented positive effects on training and negative effects on detraining for PLHA.

**Clinical Trial Registration:**www.ClinicalTrials.gov, identifier NCT03075332.

## Introduction

The perspectives of people living with HIV/AIDS (PLHA) changed dramatically after the implementation of highly active antiretroviral therapy (HAART), significantly increasing the survival of these individuals. On the other hand, this therapy promoted health complications by increasing the risk of functional decline and causing changes in body composition such as lipodystrophy (Fiorenza et al., 2011; Cardoso et al., 2013; Richert et al., 2014; Montoya et al., 2019), all of which have a negative relationship with certain components of physical fitness related to health. Other damage described in the literature report on the increase in metabolic acidosis and oxidative stress induced by the pro-inflammatory cytokines expressed by HIV that compromise airway permeability or damage lung tissues, that is, there is impairment of cardiac function (Arenas-Pinto et al., 2003; Blas-Garcia et al., 2011; Capili et al., 2011).

Given this context, supporting therapies need to be included in the daily routine of PLHA, in order to minimize these changes in infection and drug therapy. Nutritional therapies, pharmacological agents, androgen supplementation, hormone administration, and physical exercises that decrease TNF-α levels have demonstrated varying levels of success in certain parameters related to the health of HIV positive people (Dudgeon et al., 2006). Among these, physical exercise appears as a non-drug strategy, having low cost and being very effective, showing that the debilitating side effects can be practically non-existent, and are available to a much larger portion of the population.

In this sense, applications of different exercise protocols for PLHA are evidenced in the literature, these include: aerobic training (AT), resistance or strength training (RT), concurrent training (CT), and combined training (CARE), which corresponds to the combination of AT and RT (Neto et al., 2015; O’Brien et al., 2016, 2017; Kamitani et al., 2017). These protocols with two modalities applied as a combined program may be more effective in optimizing physical fitness than programs that involve only the application of one training method. Moreover, studies indicate beneficial effects of training on cardiopulmonary function (Ibeneme et al., 2019), such as improved V̇O_2_ max and muscle strength and reduced submaximal heart rate and body composition (reduced abdominal circumference, fat mass, body mass index, skinfold thickness, waist circumference, and waist/hip ratio), that is, improvement of parameters related to physical fitness and, consequently, the health of PLHA (Ezema et al., 2014; Grace et al., 2015; Leach et al., 2015; O’Brien et al., 2016; Vancampfort et al., 2018), being that these are effects that can also be found in people without HIV infection (Steib et al., 2010; Rivera-Brown and Frontera, 2012; Scribbans et al., 2016).

It is known that these benefits come from regular physical exercise, as this favors physiological adaptations to maintain the individual’s health. On the other hand, when this practice is interrupted, the organism may suffer losses from adaptations that minimize the beneficial effects of exercise, being a response to decreased physiological demand. This process is called detraining (Toraman and Ayceman, 2005; Delshad et al., 2013; Murias et al., 2015) which is a total or partial loss of adaptations induced by training (Mujika and Padilla, 2001), and can be reversed after 2–4 weeks of detraining, thus compromising physical aptitudes, and is demonstrated as being a parameter harmful to health (Sousa et al., 2019). Research seeks to study (Santos et al., 2012; Joo, 2018; Sousa et al., 2018) the effects of detraining on people without the HIV/AIDS virus, emphasizing the understanding of this process as being harmful to one’s health. However, to our knowledge, only one scientific article (Sookan et al., 2019) sought to investigate the effects of a progressive resistance training program (PRT) and the intake of whey protein on maximum muscle strength in individuals infected by the immunodeficiency virus (HIV) receiving antiretroviral therapy (ART). There were changes in maximum strength intensity after 12 weeks of interruption of PRT with continuous supplementation, but they did not elucidate the effect of detraining on other physical fitness variables in PLHA.

It must be emphasized that this population has several metabolic and physical changes, and this knowledge is important for discussions in areas such as physical training and the maintenance of the health of PLHA. In addition, a systematic review that addressed the effects of different types of exercise on health in HIV-infected patients showed that combined training showed significant gains in the results evaluated in PLHA, in this sense, through this evidence-based indication, there is the need for their application in the present study (Gomes-Neto et al., 2013).

Thus, the aim of this study was to evaluate the effect of combined training and detraining on the components of physical fitness of PLHA.

## Materials and Methods

### Study Design

This was an experimental, longitudinal study involving 15 weeks of combined training intervention and 5 weeks of detraining. The study was approved by the Research Ethics Committee of the Federal University of Rio Grande do Norte (1134038/2015) and was publicly registered (ClinicalTrials.gov Identifier: NCT03075332), meeting the items proposed by resolution 196/96-CNS-Brazil and respecting international standards for experimentation on human beings (World Medical Association, 1975).

### Participants

**FIGURE 1:**
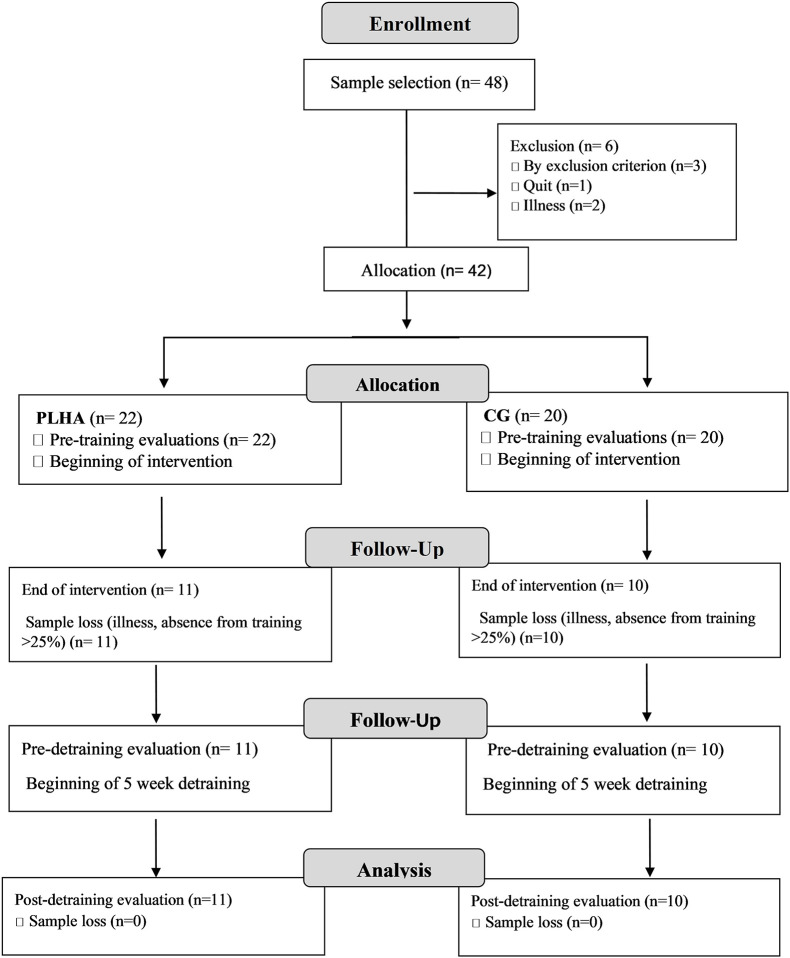
Study flowchart. PLHA, people living with HIV/AIDS group; CG, control group (people without HIV/AIDS); *n*, number of participants.

The sample consisted of 21 people divided into two groups: Group of people living with HIV (PLHA) and control group of people without HIV (CG; Figure 1). PLHA was composed of 11 people volunteered at the infectious disease hospital in the state of Rio Grande do Norte (Brazil) and undergoing clinical follow-up at the Specialized HIV/AIDS Care Service, including individuals using HAART, with TCD4 lymphocyte count equal or greater than 500 copies/mm^3^, and an undetectable viral load (≤50 copies/mm^3^). The CG was composed of 10 people without infection, selected through the use of printed posters and social media, all of whom were included without a diagnosis of HIV/AIDS or any other pathology.

For both groups, participants who were excluded had a frequency below 75% of the physical exercise program, or who had not participated in any physical exercise at the time of detraining, individuals were instructed to continue their routine of life and not to participate in any physical exercise program. Only individuals without cardiovascular disease and with a medical certificate were included. During the training period, the volunteers were monitored through telephone calls and visits to the laboratory. In addition, all participants signed an informed consent form.

#### Body Composition Assessment

This was measured by the dual energy radiological absorptiometry (DXA) method (GE Medical Systems Lunar^®^- United States), Prodigy model. The analysis of the body composition of the total body (fat mass and lean mass) and by segments (lower and upper limbs, trunk and head) was performed. The individuals were placed in the supine position, adopting the procedures described in standard protocols (Rivera-Brown and Frontera, 2012).

#### Cardiorespiratory Assessment

This was performed from a motorized treadmill stress test (Centuriom 300^®^, Brasília, Brazil), following an individualized ramp protocol, lasting between 8 and 12 min. The speed and incline were gradually increased (each minute) until the individual reached voluntary exhaustion. The same protocol was utilized during all testing sessions for each participant. Prior to the test, participants were instructed to fast for 2 h before the test.

Ventilatory variables were measured with the metabolic gas analyzer (model-Metalyzer 3B-MICROMED^®^), using the breath-by-breath method, with a 20-second window for later analysis using the Metasoft program. The Cortex unit was calibrated by the closed-loop method, using calibration gas (original 16% O_2_ and 5% CO_2_ cylinder, supplied by the manufacturer), which allowed for a new calibration before each test. The characterization of the test of reaching maximum effort was adopted based on the acceptance of at least three of the following criteria: (a) voluntary exhaustion; (b) Maximum HR reached at least 90% of that predicted for age (220-age); (c) respiratory exchange ratio > 1.1; and (d) maximum oxygen consumption, observed by the plateau concept, when V̇O_2_ stabilizes without a difference ≤ 150 ml kg^–1^ min^–1^ between the values of the last 30 s of test or peak, and when V̇O_2_ reaches a higher value during the test, with no stabilization occurring (Thompson et al., 2013; Nelson and Asplund, 2016). During the test, including rest and recovery periods, heart rate (HR) was continuously monitored using a HR monitor system (Polar^®^ FT1-Finland cardiac monitor). Blood pressure (BP) was also measured before and after the test, by the automatic Blood Pressure Monitor (Omron^®^ model HEM-7200, United States).

Specifically, the moments of the ventilatory threshold (L1), the Respiratory Compensation Point (L2), and V̇O_2_ peak were determined by three independent evaluators, using the criteria suggested by Schneider et al. (1993). The L1 was determined using the visual loss of linearity method of the relationship between oxygen consumption-V̇O_2_ and carbon dioxide-V̇CO_2_ production (V-slope; Schneider et al., 1993). To confirm the thresholds, the lowest point of the ventilatory oxygen equivalent (VE/V̇O_2_) was used, followed by its pronounced increase without a concomitant increase in the ventilatory equivalent of carbon dioxide (VE/VCO_2_), as well as the behavior of PetO_2_ and the lowest value of the respiratory exchange ratio (R).

The (L2) was analyzed from graphical inspection, where CO_2_ crossed with V̇O_2_, VE/V̇O_2_, and VE/V̇CO_2_ data. To this end, the systematic increase in ventilatory equivalents, confirmed by the higher value of PetCO_2_, preceded the abrupt decline. The V̇O_2_ peaks were the highest oxygen consumption value observed in the test (Nelson and Asplund, 2016).

#### Muscle Strength Assessment (Isometric Strength)

The isometric strength of the lower limbs was measured using a flexor/extensor chair with a coupled load cell (Miotec^®^). Thus, to analyze the strength of the flexor muscles, the participants were positioned in the chair so that the dominant leg reached the knee flexion position at range of 45°, and to analyze the strength of the extensor muscles, the participants were seated, performing a 60° knee extension (Brown et al., 2003). The strength of the upper limbs was assessed by handgrip, using the Jamar^®^ handheld dynamometer, in which the individual sat with the dominant arm at 90° elbow flexion (Sousa-Santos and Amaral, 2017). It is worth mentioning that in order to control the influence of body composition on the results of the strength test, the values of relative strength (strength correction, in kgf, and by the lean mass of the upper and lower limbs, in kg) were used.

#### Intervention Protocol

Both groups underwent CARE intervention, which consists of aerobic and resistance exercises in the same training session, respecting the training principles recommended by the American College of Sports Medicine (American College of Sports Medicine, 2013; Neto et al., 2015).

The AT was performed in a gym, lasting 20 min with walking at light intensity, controlled using the rating scale of perceived exertion (RPE; Foster, 1998). Every 2 min during the exercise, the effort scale was presented in order to keep the intensity constant. The determination of light intensity was so as to avoid exhaustion and interference in resistance training, as well as to reduce the possibility of osteoarticular injuries, characteristic of the population with HIV/AIDS (Yahiaoui et al., 2012; O’Brien et al., 2016).

Resistance training occurred immediately after AT, being prescribed based on the flexible non-linear periodization model. Through this method, study volunteers could self-select the training load immediately before training, based on their physiological preparedness (McNamara and Stearne, 2013), and it also involves the variation of stimuli through changes in training intensity each week (American College of Sports Medicine, 2013; Prestes et al., 2015). The variation occurred through stimuli of resistance, hypertrophy and strength. The periodization was divided into a total period of 15 weeks (Macrocycle), being subdivided into five mesocycles of 3 weeks (each week had a specific microcycle: resistance, hypertrophy, and strength). Before starting the resistance training sessions, the volunteers were reminded of the load lifted previously, respective to the same stimulus session. Then they were motivated to increase the load keeping to the prescribed repetitions and the suggested perceived exertion. If the perceived exertion increased, the load could be adjusted. The intensity of resistance training was monitored by the OMNI-res scale (Lagally and Robertson, 2006). It is worth mentioning that the prescription used in the study sought to emphasize exercises for the body segments most affected by lipodystrophy, respecting a stimulus interval between sessions of 48 h during the week and 72 h at the weekend. This CARE intervention aimed to recover the muscular damage of the participants and to avoid accentuated immunosuppression (Pedro et al., 2016).

During the training and detraining protocol, the volunteers were instructed to maintain their eating habits.

After the completion of the 15 weeks training intervention, both groups were evaluated and then the detraining period was performed, characterized by the suspension of training for 5 weeks. All participants were advised not to perform any type of physical exercise during this period, maintaining only normal daily activities.

#### Statistical Treatments

The Shapiro–Wilk and Levene tests were performed to analyze the data distribution and homogeneity, respectively, and all assumptions were met. Thus, the results presented in the tables are expressed as mean and standard deviation (SD). To compare the descriptive characteristics of the PLHA and CG (Table 1), an independent samples *t*-test was conducted.

**TABLE 1:** Training intervention protocol.

	**Aerobic training**	**Resistance training**
Frequency	3× weekly (non-consecutive days)	3× weekly (non-consecutive days)
Duration	20 min/session	60–80 min/session
Intensity	Low (4–5 BORG)	*Weekly Undulatory* Resistance: RPE 4-6 OMNI-Res Hypertrophy: RPE 7-8 OMNI-Res Strength: RPE 8-10 OMNI-Res
Volume	Constant	*6 exercises/session* Resistance: 3 × 15 repetitions Hypertrophy: 3 × 11 repetitions Strength: 3 × 6 repetitions
Rest interval	None	Resistance: 60 s Hypertrophy: 90 s Strength: 150 s
Method	Continuous	(A) Quadriceps, chest and abdomen (upper) (B) Hamstring, dorsal, calf and abdomen (lower) (C) Triceps, biceps, shoulder. Gluteus and abdomen (transverse and oblique)

A 2 × 3 mixed-model ANOVA with repeated measurements for group (between) and time (within) was used to examine the effects of the intervention (training and detraining) on the variables of body composition, strength (handgrip and isometric) and cardiorespiratory fitness. The results of the significance level and the effect size (partial eta squared – $\eta_{p}^{2}$) are presented. For significant time or interaction (time vs. group) effects, the Bonferroni *post-hoc* test was adopted to locate the source of the differences. The assumption of sphericity was assessed with the Mauchly test, and the Greenhouse–Geisser epsilon correction was applied when significant violations were found. All analyzes were performed using the GraphPad Prism 6.0 and SPSS Inc. 22.0 software, adopting a significance level of *p* < 0.05.

## Results

Seeking to understand the adaptations to physical training, the variables of physical fitness (body composition, cardiorespiratory function, and strength) of the participants were evaluated at three moments of the study: pre-training (Pre-TR); post-training (Pos-TR); and post-detraining (Post-DTR), occurring after 5 weeks of interruption of physical exercise. All assessments took place at the same time, in the morning, adopting an interval of 48 h between the assessments of body composition, cardiorespiratory function and strength. In addition, the participants were instructed to abstain from caffeine, smoking, and physical exercise in the 24 h prior to the tests.

Table 2 presents the description of the study participants, demonstrating that there is homogeneity between the characteristics of the groups, with the exception of body weight.

**TABLE 2:** Descriptive characteristics of the PLHA and CG.

	**PLHA**	**CG**	***P***
Age (year)	46.9 ± 8.0	43.8 ± 13.8	0.54
Weight (kg)	63.8 ± 12.7	75.2 ± 11.2	0.04
Height (cm)	161.7 ± 8.7	163.3 ± 7.8	0.66
BMI (Kg/*m*^2^)	24.2 ± 3.0	28.3 ± 4.7	0.03
M/W (n)	7/4	3/7	–
Medication	HAART	None	–
IPAQ	Irregularly active	Irregularly active	–

*PLHA, people living with HIV/AIDS group; CG, control group (people without HIV/AIDS); M, men; W, women; IPAS, International Physical Activity Survey; HAART, highly active antiretroviral therapy (protease inhibitors and reverse-transcriptase inhibitors and no nucleotide).*

Table 3 presents the results of body composition, according to the Pre-TR, Pos-TR, and Post-DTR moments of the PLHA and CG groups. The time factor was significant for the percentage of fat in the segments of the leg (*F*_2,38_ = 3.335; *p* = 0.046; $\eta_{p}^{2}=0.149$), trunk (*F*_2,38_ = 4.571; *p* = 0.017; $\eta_{p}^{2}=0.194$) and percentage of total fat (*F*_2,38_ = 4,161; *p* = 0.023; $\eta_{p}^{2}=0.180$). From the analysis of the *post hoc* test, it was possible to identify an overall effect of training on the percentage of fat of the leg (−3.3%; *p* = 0.033), trunk (−2.9%; *p* = 0.049), and total fat (−2.8%; *p* = 0.007). There was also an overall effect of detraining on the percentage of trunk fat (+2.4%; *p* = 0.009). However, no significant differences were identified in the group time interaction.

**TABLE 3:** Descriptive and inferential results of body composition variables pre-training (Pre-TR); post-training (Pos-TR); and post-detraining (Post-DTR) for PLHA and CG.

	**Pre-TR**	**Post-TR**	**Post-DTR**	**Time Effect**		**Interaction**	
				***F***	***p***	***F***	***p***
**Body mass, Kg**
PLHA	63.8 ± 12.7	62.8 ± 10.6	62.8 ± 14.3	0.142	0.868	0.080	0.924
Control group	75.2 ± 11.2	75.0 ± 12.4	75.2 ± 12.5				
**Fat-free mass, Kg**
PLHA	44.7 ± 9.6	45.6 ± 9.7	45.1 ± 10.0	3.088	0.080	0.047	0.894
Control group	48.1 ± 11.5	48.8 ± 11.5	48.7 ± 11.6				
**Arm fat, %**	
PLHA	24.5 ± 9.8	23.7 ± 9.6	23.7 ± 9.6	0.908	0.412	1.194	0.314
Control group	35.3 ± 12.8	34.6 ± 12.6	34.3 ± 12.7				
**Leg fat, %**
PLHA	24.9 ± 9.5	24.7 ± 9.7	23.6 ± 9.8	3.335	0.046	2.056	0.142
Control group	36.4 ± 10.6	34.7 ± 10.4	35.0 ± 10.8				
**Trunk fat, %**
PLHA	31.1 ± 8.2	31.0 ± 7.7	31.0 ± 8.8	4.571	0.017	3.073	0.074
Control group	37.7 ± 12.8	36.0 ± 12.6	36.7 ± 12.3				
**Total fat, %**
PLHA	27.6 ± 7.5	27.5 ± 7.3	27.2 ± 7.9	4.161	0.023	2.412	0.103
Control group	35.9 ± 11.3	34.5 ± 11.1	34.9 ± 11.2				

*PLHA, people living with HIV/AIDS group; CG, control group (people without HIV/AIDS).*

*Interaction = group vs. time interaction two-way repeated measures ANOVA.*

In regard to Table 4, significant differences were found in the time factor for all Handgrip and isometric strength variables: Right Handgrip strength (*F*_2,38_ = 4.534; *p* = 0.017; $\eta_{p}^{2}=0.193$), Left Handgrip strength (*F*_2,38_ = 3.655; *p* = 0.035; $\eta_{p}^{2}=0.161$), Left Flexion MVIS (*F*_2,38_ = 9.203; *p* = 0.005; $\eta_{p}^{2}=0.326$), Right Flexion MVIS (*F*_2,38_ = 7.485; *p* = 0.008; $\eta_{p}^{2}=0.283$), Left Extension MVIS (*F*_2,38_ = 5.938; *p* = 0.006; $\eta_{p}^{2}=0.238$), and e Right Extension MVIS (*F*_2,38_ = 10.316; *p* = <0.001; $\eta_{p}^{2}=0.352$).

**TABLE 4:** Descriptive and inferential results of Handgrip and isometric strength (Pre-TR, Pos-TR, and Post-DTR) for PLHA and CG.

	**Pre-TR**	**Post-TR**	**Post-DTR**	**Time effect**		**Interaction**	
				***F***	***p***	***F***	***p***
**Right handgrip strength, Kgf/Kg**
PLHA	13.05 ± 2.52	13.69 ± 2.56	13.48 ± 2.77	4.534	0.017	0.108	0.898
Control group	12.05 ± 1.47	12.92 ± 1.52	12.51 ± 1.24				
**Left handgrip strength, Kgf/Kg**
PLHA	13.07 ± 1.70	13.66 ± 1.13	13.71 ± 1.84	3.655	0.035	0.189	0.829
Control group	12.05 ± 1.60	13.03 ± 1.53	12.80 ± 1.59				
**Left flexion MVIS, Kgf/Kg**
PLHA	1.97 ± 0.29	2.36 ± 0.38	2.24 ± 0.40	9.203	0.005	0.032	0.969
Control group	1.89 ± 0.64	2.31 ± 0.34	2.22 ± 0.32				
**Right flexion MVIS, Kgf/Kg**
PLHA	1.87 ± 0.55	2.51 ± 0.42	2.27 ± 0.44	7.485	0.008	2.178	0.150
Control group	2.02 ± 0.33	2.20 ± 0.24	2.19 ± 0.37				
**Left extension MVIS, Kgf/Kg**
PLHA	4.97 ± 0.92	5.48 ± 1.02^*a*^	4.81 ± 1.08^*b*^	5.938	0.006	4.429	0.019
Control group	4.79 ± 1.29	5.12 ± 1.22	5.25 ± 1.05				
**Right extension MVIS, Kgf/Kg**
PLHA	4.99 ± 0.92	5.56 ± 0.93^*a*^	4.85 ± 1.05^*b*^	10.316	< 0.001	3.848	0.030
Control group	4.40 ± 1.23	4.81 ± 1.10	4.72 ± 0.86				

*The absolute values of strength were corrected by the Fat-free mass (Kg) of the arm (handgrip strength) and leg (MVIS). Kg, kilogram; Kgf, kilogram force.*

*PLHA, people living with HIV/AIDS group; CG, control group (people without HIV/AIDS); MVIS, maximal voluntary isometric strength.*

*Interaction = group vs. time interaction two-way repeated measures ANOVA*

*^*a*^Significant difference compared to the Pre-TR, HIV group (training effect, *p* < 0.05).*

*^*b*^Significant difference compared to the Post-TR, HIV group (detraining effect, *p* < 0.05).*

As for the overall training effect, significant mean differences were found for the variables of Left Flexion MVIS (+21.1%, *p* = 0.006), Right Flexion MVIS (+21.6%, *p* = 0.009), Left Extension MVIS (+8.6%, *p* = 0.001), and Right Extension MVIS (+10.5%, *p* = 0.001). In regard to the general effect of detraining, significant differences were found only for the variables of Left Flexion MVIS (−4.2%, *p* = 0.032) and Right Extension MVIS (−7.8%, *p* = 0.013).

Furthermore, significant differences in the interaction factor (group vs. time) were found for the variables of Left Extension MVIS (*F*_2,38_ = 4.429; *p* = 0.019; $\eta_{p}^{2}=0.189$) and Right Extension MVIS (*F*_2,38_ = 3.848; *p* = 0.030; $\eta_{p}^{2}=0.168$). These findings demonstrate a significant increase in isometric extension strength during the training period (left: 10.4%; right: 12.4%) and a subsequent significant decrease in the detraining period (left: −10.8%; right: −12.1%) in the PLHA group compared with the control group (left: 8.1 and 3.9%, respectively; right: 11.5 and −0.2%, respectively).

The results of oxygen consumption are shown in Table 5. Considering the different intervention moments (time factor), significant differences were observed for V̇O_2_ VT1 (*F*_2,38_ = 24,386; *p* < 0.001; $\eta_{p}^{2}=0.562$), V̇O_2_ VT2 (*F*_2,38_ = 16,176; *p* < 0.001; $\eta_{p}^{2}=0.460$), and V̇O_2_ Peak (*F*_2,38_ = 19,774; *p* < 0.001; $\eta_{p}^{2}=0.510$), with average differences in general training of +23.2% (*p* < 0.001), +19.7% (*p* < 0.001), and 18.3% (*p* < 0.001), respectively. In addition, general detraining demonstrated mean differences of −17.0% (*p* < 0.001), −14.6% (*p* < 0.001), and −8.2% (*p* < 0.001), respectively.

**TABLE 5:** Descriptive and inferential results of oxygen uptake (Pre-TR, Pos-TR, and Post-DTR) for PLHA and CG.

	**Pre-TR**	**Post-TR**	**Post-DTR**	**Time effect**		**Interaction**	
				***F***	***p***	***F***	***p***
**V̇O_2_ VT1, ml kg min^–1^**
PLHA	14.55 ± 2.66	18.42 ± 3.84^*a*^	13.91 ± 2.12^*b*^	24.386	<0.001	7.261	0.002
Control group	11.3 ± 1.77	13.43 ± 1.50^#^	12.53 ± 1.73				
**V̇O_2_ VT2, ml kg min^–1^**
PLHA	25.09 ± 6.19	30.56 ± 6.36	25.91 ± 4.41	16.176	<0.001	0.177	0.839
Control group	24.60 ± 6.08	28.95 ± 7.74	24.90 ± 6.79				
**V̇O_2_ Peak, ml kg min^–1^**
PLHA	29.82 ± 5.02	33.96 ± 6.05	31.27 ± 6.36	19.774	<0.001	0.998	0.361
Control group	26.70 ± 6.36	32.92 ± 9.35	30.10 ± 8.61				

*PLHA, people living with HIV/AIDS group; CG, control group (people without HIV/AIDS); V̇O_2_, oxygen uptake; VT1, ventilatory threshold 1; VT2, ventilatory threshold 2.*

*Interaction = group vs. time interaction two-way repeated measures ANOVA.*

*^*a*^Significant difference compared to the Pre-TR, HIV group (training effect, *p* < 0.05).*

*^*b*^Significant difference compared to the Post-TR, HIV group (detraining effect, *p* < 0.05).*

*^#^Significant difference compared to the Pre-TR, CG group (training effect, *p* < 0.05).*

Furthermore, there was a significant difference for the interaction factor (group vs. time) in V̇O_2_ VT1 (*F*_2,38_ = 7.261; *p* = 0.002; $\eta_{p}^{2}=0.277$), indicating a significantly greater increase with training (27.3%) and decrease with detraining (−22.7%) in the PLHA group compared with the Control group (19.9 and −6.7%, respectively).

## Discussion

The aim of our study was to evaluate the effect of training and detraining on the components of physical fitness of PLHA compared to uninfected individuals (CG). Given this purpose, our main finding was that 15 weeks of training promoted improvements in cardiorespiratory fitness, specifically related to ventilatory threshold 1, and in lower limb extension strength in both groups, while it was also found that detraining promoted significant decreases in the same variables after 5 weeks of suspension of training in PLHA, when compared to uninfected individuals.

Cardiorespiratory capacity, measured by V̇O_2_, is an important predictor of cardiovascular diseases, directly related to mortality in the general population (Steib et al., 2010). Improvements above 10% after a training program are known to be clinically significant in patients with chronic disease. Our results showed improvement after a combined training program in all parameters (thresholds 1, 2 and peak V̇O_2_), which is also observed in interventions with exercise programs for people without HIV (Frankenstein et al., 2007).

Among the variables of cardiorespiratory fitness analyzed, ventilatory threshold 1, characterized as the limit between the predominantly aerobic exercise phase and the phase that starts metabolic acidosis or where the transition of muscle energy production and increase in blood lactate concentration occur (Maté-Muñoz et al., 2015), was the one with the greatest reductions in PLHA due to the suspension of training. These damages can be explained by the reduced physiological demand, which causes lower mitochondrial and capillary density, which consequently reduces the oxygenation of the muscle tissue in the peripheries (Mujika and Padilla, 2001).

A probable explanation for this cardiorespiratory deficit is due to a specific behavior in PLHA elucidated in the literature, which refers to metabolic acidosis, resulting from oxidative stress. This originates from the action of the virus itself in the body and the toxicity of some antiretroviral drugs, which produce excess reactive oxygen species (ROS) and reach CD4+ T cells, causing a decline in the antioxidant capacity, making it more difficult for the individual to achieve redox balance, consequently causing cell damage in the mitochondria, one of the most important cell organelles responsible for transporting electrons to the respiratory chain (Blas-Garcia et al., 2011; Vassimon et al., 2012; Perez-Matute et al., 2013).

Detraining, therefore, seems to emphasize the mechanisms that are already present in PLHA, and generally hinders the benefits that regular combined training promotes, such as the improved effect on post-exercise excessive oxygen consumption (PEOC), an action that allows for the removal of metabolics, thus decreasing lactic acidosis. As for threshold 2 and V̇O_2_ peak, our findings show that detraining did not cause significant losses. This may be due to the fact that the impact of other variables that might influence the adaptation to training (such as basal metabolic rate, hormones, and cytokines, etc.,) were not evaluated in this study, but the importance of these results is recognized, and therefore it would be interesting to evaluate these in future studies.

It has already been reported that people without HIV present loss of cardiorespiratory fitness with the suspension of physical exercise (Lovell et al., 2011; Grace et al., 2015). However, we did not find studies that present the effects of detraining in PLHA, which is a knowledge gap in this discussion, but also shows that there needs to be an important investigation that aims to fill this gap.

In the muscle strength variable, it was observed that training promoted improvements in strength in the lower limbs and detraining caused greater declines in the extension strength of lower limbs in the PLHA group. As for handgrip strength, there was no change during training and detraining in both groups. We propose that these differences between upper and lower body strength occurred because of the smaller muscle mass involved in the upper body in training, which would produce smaller gains. Previous researchers have also observed this phenomenon when improvements in the lower body were greater than those in the upper body, regardless of the training program used (Kok et al., 2009).

A possible explanation for the effect of detraining on PLHA is due to the fact that these subjects have lipoatrophy (change in body composition with loss of fat in the peripheral regions) as an adverse effect, which is directly related to sarcopenia (loss of muscle mass), as reported by Raso et al. (2013) which points to a reduction in muscle mass and the consequent loss of muscle strength in PLHA due to the virus (Sousa-Santos and Amaral, 2017).

In regard to for body composition, we observed that both training and detraining did not cause significant differences. This may be due to the intervention short training duration time (15 weeks). In PLHA, studies report the difficulty in significantly modifying the body composition of this population in different intervention periods (Foster, 1998; Dudgeon et al., 2006).

The scarcity of studies that have evaluated the effect of detraining on PLHA makes it difficult to compare the findings, but, at the same time, a strong point of our analysis is the originality and relevance of the results presented in this study. On the other hand, some limitations can be seen, such as the small sample size, a recurrent case in studies with populations with HIV and with medium-term intervention, as well as measurements of biochemical, protein and/or gene markers that confirm the justification that oxidative stress and metabolic acidosis are caused by greater detraining.

Therefore, the study demonstrated that combined training and detraining caused similar overall responses between PLHA and uninfected individuals, with regard to body composition, isometric strength, and cardiorespiratory fitness. However, the ventilatory threshold 1 (oxygen consumption) and the extension strength of the lower limbs showed significant changes in the PLHA group when compared to uninfected individuals, causing a positive effect of training and, later, deleterious effects of the same variables after 5 weeks suspension from training.

Given all these findings, it is suggested that the suspension of physical exercise programs be avoided, especially in PLHA, since the effects of detraining were more pronounced in this population. It is also essential to encourage this public to practice exercise constantly, as a measure of prevention and treatment of cardiometabolic diseases.

## Data Availability Statement

The original contributions presented in the study are included in the article/supplementary material, further inquiries can be directed to the corresponding author.

## Ethics Statement

The studies involving human participants were reviewed and approved by Comitê de Ética e Pesquisa da Universidade Federal do Rio Grande do Norte. The patients/participants provided their written informed consent to participate in this study.

## Author Contributions

JA designed the study and provided guidance throughout the research process and possesses expertise in HIV and exercise research. RM, TS, DM, JM, RM, and PW developed the protocol, collected and analyzed the data, and drafted the manuscript. JA, IS, BC, and PD were closely reviewed and guided all steps. All authors read and approved the final manuscript.

## Conflict of Interest

The authors declare that the research was conducted in the absence of any commercial or financial relationships that could be construed as a potential conflict of interest.

## Publisher’s Note

All claims expressed in this article are solely those of the authors and do not necessarily represent those of their affiliated organizations, or those of the publisher, the editors and the reviewers. Any product that may be evaluated in this article, or claim that may be made by its manufacturer, is not guaranteed or endorsed by the publisher.
